# Early treatment interruption and nutritional status as predictors of mortality in *Mycobacterium avium* complex pulmonary disease

**DOI:** 10.1371/journal.pone.0350106

**Published:** 2026-05-27

**Authors:** Hye Young Hong, Youngmok Park, Song Yee Kim, A La Woo, Hye-Jeong Lee, Young Ae Kang

**Affiliations:** 1 Division of Pulmonary and Critical Care Medicine, Department of Internal Medicine, Yonsei University College of Medicine, Seoul, Republic of Korea; 2 Department of Radiology, Research Institute of Radiological Science, Yonsei University College of Medicine, Seoul, Republic of Korea; Showa University Fujigaoka Hospital, JAPAN

## Abstract

*Mycobacterium avium* complex pulmonary disease (MAC-PD) requires prolonged multidrug therapy. However, treatment outcomes remain suboptimal due to limited antibiotic efficacy, disease chronicity, and frequent early treatment interruption (ETI). Despite its clinical significance, data on the prevalence, risk factors, and long-term outcomes of ETI remain limited. Therefore, this study aims to identify factors associated with ETI and its impact on mortality. A retrospective cohort study of 420 patients treated with MAC-PD was conducted between 2010 and 2023. Patients were categorized into ETI (treatment duration < 12 months) and standard groups (treatment duration ≥ 12 months). Logistic regression was used to identify risk factors for ETI, whereas multivariate Cox proportional hazards regression was employed to evaluate factors associated with mortality. ETI occurred in 30% of patients. Low prognostic nutritional index (PNI) (adjusted odds ratio [aOR]: 0.92; 95% confidence Interval [CI]: 0.87–0.99) and grade 2 or higher adverse drug reactions (ADRs) (aOR: 5.65; 95% CI: 2.45–15.00) increased the risk of ETI. ETI was associated with higher mortality (adjusted hazard ratio: 2.86; 95% CI: 1.51–5.40). Additionally, low PNI scores indicated increased mortality risk. ETI is prevalent in MAC-PD and is strongly associated with ADRs and poor nutrition, both of which also predict higher long-term mortality. Early ADR monitoring and nutritional support are essential for improving treatment adherence and patient outcomes.

## Introduction

The treatment of *Mycobacterium avium* complex pulmonary disease (MAC-PD) presents significant clinical challenges. Effective management of MAC-PD requires a multidrug regimen, typically including macrolides, ethambutol, and rifamycins, administered for at least 12 months after microbiologic culture conversion [1]. Despite these prolonged and intensive regimens, treatment success rates remain suboptimal, with only 33–68% of patients achieving long-term microbiological cure, depending on disease severity and the presence of comorbid conditions [2,3].

Current guidelines recommend continuing therapy for at least 12 months after achieving culture conversion to optimize treatment success. However, treatment intolerance encounters a significant barrier to this goal, as many patients experience adverse drug reactions (ADRs), such as gastrointestinal disturbances, hepatotoxicity, neurologic issues, and hypersensitivity, often leading to premature treatment discontinuation [4–7]. These early treatment interruptions (ETI) may hinder effective disease control and negatively impact clinical outcomes [8].

Despite these challenges, few studies have focused on the intolerability of antimycobacterial treatment or the long-term outcomes of patients who experience pre-term cessation of therapy [9]. Most studies have focused primarily on the clinical outcomes of patients who complete the standard 12-month regimen [10,11], leaving a critical gap in understanding the implications of early discontinuation of treatment.

Given the limited data on patients who discontinue treatment before completing the standard 12-month duration after culture conversion, this study aims to identify factors associated with ETI, defined as the cessation of antimycobacterial therapy within 12 months. Additionally, the study evaluates the long-term outcomes of patients with MAC-PD who stop treatment prematurely, providing insights into the impact of ETI on mortality.

## Materials and methods

### Study population

**Fig 1:**
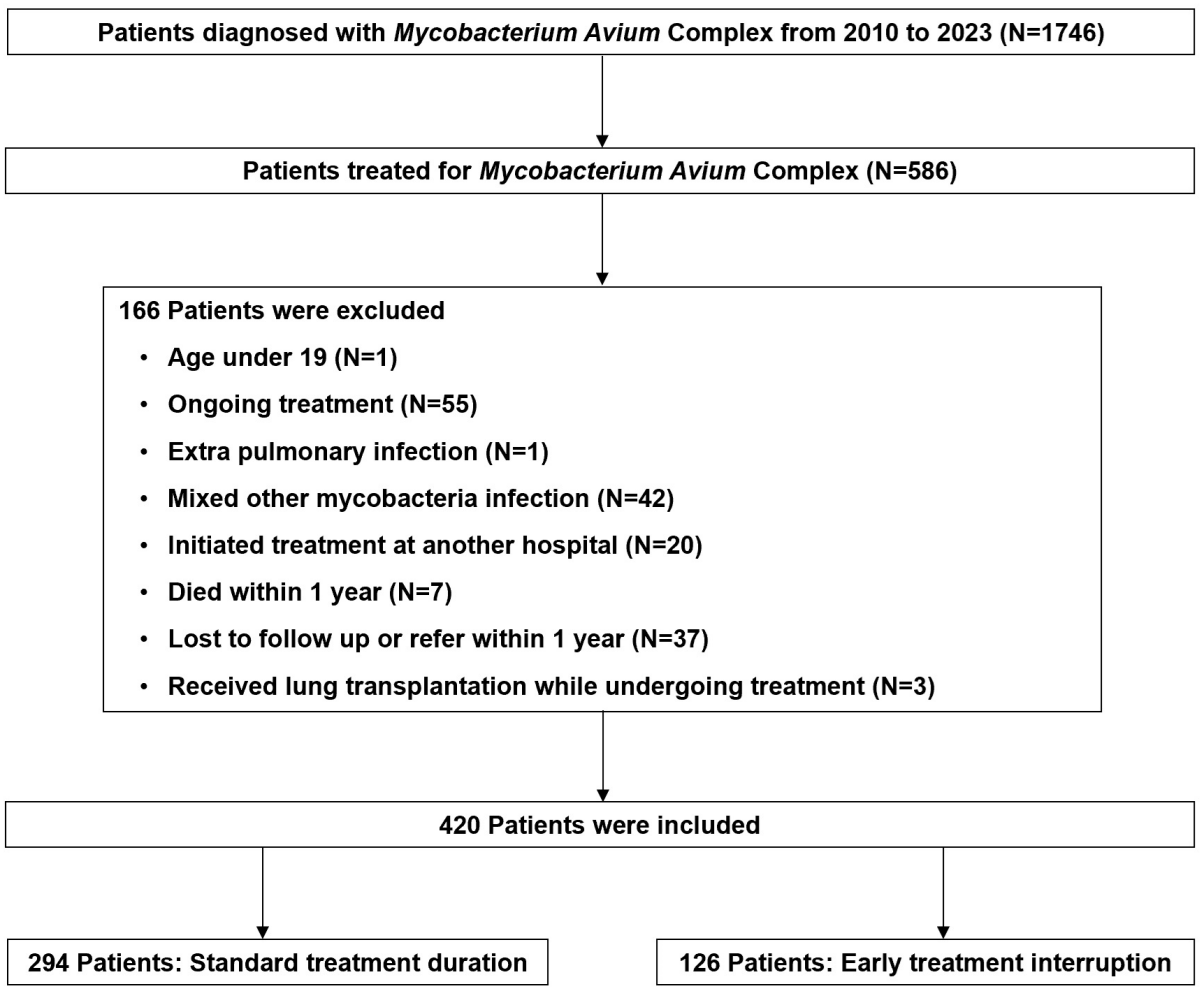
Flow chart.

We conducted a retrospective cohort study of adults treated with MAC-PD between 2010 and 2023, including only cases that met the 2007 American Thoracic Society and the Infectious Diseases Society of America criteria for nontuberculous mycobacterial pulmonary disease (NTM-PD) [12]. The anonymous data were accessed for research purposes between July 1, 2024, and May 30, 2025. Exclusion criteria were patients younger than 19 years, those receiving ongoing treatment, individuals with extrapulmonary or mixed mycobacterial infections, patients who initiated treatment at other institutions, and those who died, were lost to follow-up, transferred, or underwent lung transplantation within one year of treatment initiation (Fig 1).

### Data collection

Demographic and clinical data, including age, sex, body mass index (BMI), smoking history, previous tuberculosis history, comorbidities, initial symptoms, radiological findings, and mycobacterial Acid-Fast Bacillus (AFB) smear and culture results, were collected. Laboratory data were collected to assess baseline immunonutritional status using the Prognostic Nutritional Index (PNI), originally proposed for surgical risk assessment [13], PNI is a simple, low-cost immunonutritional marker calculated from serum albumin and total lymphocyte count as follows: (10 × serum albumin [g/dL]) + (0.005 × total lymphocyte count [/mm³]). Serum albumin reflects both nutritional status and systemic inflammation, while the total lymphocyte count represents immune competence. Based on the original criteria proposed by Onodera et al., PNI < 45 was considered indicative of increased clinical risk [14].

We recorded treatment-related ADRs, their affected organ systems (as defined by the Medical Dictionary for Regulatory Activities [15], and severity (categorized using the Common Terminology Criteria for Adverse Events [CTCAE]) [16]. ADRs were identified through systematic review of electronic medical records, including clinician documentation, laboratory abnormalities, and relevant diagnostic test results. Mortality during follow-up was ascertained using hospital medical records and through linkage with the national death registry provided by Statistics Korea. All linked data were anonymized before being provided to the investigators for analysis.

### Definition

Treatment outcomes of NTM-PD were classified according to the NTM-NET consensus statement criteria [17]. Culture conversion was three or more consecutive negative cultures from respiratory samples, collected ≥4 weeks apart during treatment. Treatment success included cure and clinical cure. Treatment failure was defined as the absence of culture conversion after at least 12 months of appropriate antimycobacterial therapy. Treatment halted was defined as the premature cessation of antimycobacterial therapy initiated by either the physician or the patient. Lost to follow-up (LTFU) was defined as the interruption of treatment for two or more consecutive months due to missed follow-up visits.

Patients were categorized into two groups: the standard treatment group (defined as patients who completed ≥12 months of prescribed MAC-PD treatment) and the ETI group (treatment duration <12 months).

### Statistical analysis

Categorical variables are presented as numbers and percentages, and continuous variables are expressed as means with standard deviations (SD) or medians with interquartile ranges (IQR). Group comparisons were performed using the chi-square test. Logistic regression analysis was used to identify risk factors for ETI, with covariates showing P < 0.05 in the univariate model included in the multivariate analysis. Multicollinearity among variables was assessed using the Variance Inflation Factor. Age, sex, and BMI were included as common adjustment variables, and PNI was prioritized to avoid multicollinearity with albumin and lymphocyte levels. For survival analysis, we used Cox proportional hazards regression. Statistical significance was set at P < 0.05, and all analyses were conducted using R software (version x64 4.4.1, R Foundation for Statistical Computing, Vienna, Austria).

### Ethics statements

The study protocol was reviewed and approved by the Institutional Review Board (IRB) of the Severance Hospital Ethics Committee (IRB approval number: 4-2024-0574). The requirement for informed consent was waived due to the retrospective nature of the study.

## Results

### Baseline characteristics of study populations

As illustrated in the study flow diagram (Fig 1), a total of 586 patients were initially screened, 166 were excluded based on the predefined criteria, resulting in a final analytic cohort of 420 patients.

The baseline characteristics of the 420 patients who initiated treatment for MAC-PD are summarized in Table 1. The median age was 65 years, with 37.4% (N = 157) being male. The mean BMI was 20.3 kg/m², and sputum production was the most frequently reported symptom (51.4%). Positive sputum AFB smear results were observed in 22.4% of patients. Radiologically, the nodular bronchiectatic form without cavity was the most prevalent type. The median PNI score at treatment was 50.9 (IQR 46.6–54.2).

**Table 1:** Clinical characteristics of patient with MAC-PD based on treatment duration.

Characteristics	Total	Standard group	ETI	*P* value
	(N = 420)	(N = 294)	(N = 126)	
Age (years)	65 (57–73)	63 (56–72)	68 (60–75)	0.002
Sex				0.103
Male	157 (37.4)	102 (34.7)	55 (43.7)	
Female	263 (62.6)	192 (65.3)	71 (56.3)	
BMI (kg/m^2^)	20.3 ± 3.0	20.2 ± 2.9	20.4 ± 3.3	0.698
Smoking history				0.520
Non-smoker	317 (75.5)	225 (76.5)	92 (73.0)	
Ever-smoker	103 (24.5)	69 (23.5)	34 (27.0)	
Comorbidities				
History of pulmonary TB	169 (40.2)	121 (41.2)	48 (38.1)	0.633
Diabetes	55 (13.1)	38 (12.9)	17 (13.5)	1.000
Interstitial lung disease	8 (2.7)	6 (3.0)	2 (2.1)	0.949
COPD	58 (13.8)	38 (12.9)	20 (15.9)	0.517
Asthma	28 (6.7)	21 (7.1)	7 (5.6)	0.701
Malignancy	75 (17.9)	45 (15.3)	30 (23.8)	0.052
Cardiovascular disease	55 (13.1)	33 (11.2)	22 (17.5)	0.115
Chronic kidney disease	19 (4.5)	13 (4.4)	6 (4.8)	1.000
Connective tissue disease	20 (4.8)	13 (4.4)	7 (5.6)	0.803
Chronic liver disease	25 (6.0)	19 (6.5)	6 (4.8)	0.653
Symptom presence at initial visit				
Cough	189 (45.0)	129 (43.9)	60 (47.6)	0.549
Sputum	216 (51.4)	156 (53.1)	60 (47.6)	0.360
Dyspnea	46 (11.0)	32 (10.9)	14 (11.1)	1.000
Hemoptysis	80 (19.0)	60 (20.4)	20 (15.9)	0.343
Others^a^	52 (12.4)	38 (12.9)	14 (11.1)	0.722
Sputum AFB smear results, positive	94 (22.4)	68 (23.1)	26 (20.6)	0.664
Radiologic type				0.469
FC type	37 (8.8)	29 (9.9)	8 (6.3)	
NB type with cavity	111 (26.4)	79 (26.9)	32 (25.4)	
NB type without cavity	258 (61.4)	178 (60.5)	80 (63.5)	
Others^b^	14 (3.3)	8 (2.7)	6 (4.8)	
Hemoglobin (g/dL)	13.0 (12.1–13.9)	13.1 (12.2–14.0)	12.8 (11.9–13.6)	0.022
PNI, at treatment	50.9 (46.6–54.2)	51.6 (47.8–54.7)	47.9 (43.1–52.7)	<0.001
BACES score^c^ (N = 36)	3.0 (1.0–3.0)	2.5 (1.0–3.0)	3.0 (1.0–4.0)	0.789

Data are presented as numbers (percentages), mean (standard deviation), median (interquartile range).

ETI = early treatment interruption, BMI = body mass index, TB = tuberculosis, COPD = chronic obstructive pulmonary disease, AFB = acid fast bacillus, FC = fibro-cavity, NB = nodular bronchiectasis, BACES = BMI, age, cavity, erythrocyte sedimentation rate, and sex, PNI = prognostic nutritional index, MAC-PD = *Mycobacterium avium* complex pulmonary disease.

^a^Others include fever, weight loss, and general weakness.

^b^Others include consolidation, nodule only.

^c^BACES score is a validated prognostic scoring system for mortality in patients with NTM-PD. It comprises five components: body mass index (<18.5 kg/m²), age (≥65 years), presence of cavity on imaging, elevated erythrocyte sedimentation rate (>15 mm/h in men, > 20 mm/h in women), and male sex. Higher scores indicate an increased risk of long-term mortality.

ETI occurred in 30% (n = 126) of patients, indicating treatment cessation within 12 months. Among these 126 patients, 15 discontinued therapy despite not experiencing any ADRs (6 patients had patient-initiated interruptions, and 9 had physician-initiated interruptions based on underlying comorbidities and other clinical considerations). Regarding the timing of ETI, 34% discontinued treatment within the first 2 months, and 63% within 6 months (S1 Fig). Comparisons between the ETI and the standard treatment groups revealed that patients in the ETI group were older (68 vs 63 years), and had lower and PNI scores (47.9 vs 51.6) at treatment initiation.

### Adverse drug reactions

ADRs were common across the study population, with an overall prevalence of 68.8%. (S1 Table) However, the ETI group showed a higher incidence of ADRs (88.1%) compared with the standard group (60.5%). Additionally, the proportion of grade 2 or higher ADRs was significantly higher in the ETI group (81.0% vs 38.1%). The median time to ADR onset in the ETI group was 36 days, which was shorter compared with the standard group (41 days). Furthermore, 29.4% of patients in the ETI group experienced ADRs involving two or more organ systems.

When analyzed by organ system, gastrointestinal disorders were the most common ADRs, followed by nervous system disorders, eye disorders, and skin disorders (Table 2). These findings suggest that the frequency, severity, and multi-organ involvement of ADRs may markedly contribute to early treatment interruption in MAC-PD.

**Table 2:** Adverse drug reactions during MAC-PD treatment.

Characteristics	Standard group	Early treatment interruption	*P* value
	(N = 294)	(N = 126)	
Any adverse drug reactions	N = 178	N = 111	
Grade of adverse drug reactions			<0.001
Grade 1	66 (37.1)	9 (8.1)	
Grade 2	110 (61.8)	95 (85.6)	
Grade 3 or higher	2 (1.1)	7 (6.3)	
Organ specific adverse drug reactions			
Blood and lymphatic system disorders	7 (2.4)	7 (5.6)	0.172
Metabolism and nutrition disorders	12 (4.1)	6 (4.8)	0.958
Nervous system disorders	12 (4.1)	17 (13.5)	0.001
Eye disorder	41 (13.9)	17 (13.5)	1.000
Ear and labyrinth disorders	19 (6.5)	6 (4.8)	0.653
Cardiac disorders	1 (0.3)	5 (4.0)	0.015
Gastrointestinal disorders	78 (26.5)	56 (44.4)	<0.001
Hepatobiliary disorders	14 (4.8)	4 (3.2)	0.636
Skin and subcutaneous tissue disorders	27 (9.2)	14 (11.1)	0.667
Renal and urinary disorders	2 (0.7)	3 (2.4)	0.326
General disorders	5 (1.7)	10 (7.9)	0.004
Others^a^	9 (3.1)	11 (8.7)	0.024

MAC-PD = *Mycobacterium avium* complex pulmonary disease.

Data are presented as numbers (percentages).

^a^Others include dry mouth, mood change, myalgia.

### Risk factors for early treatment interruption of MAC-PD

Logistic regression analysis was used to identify key risk factors associated with ETI (Table 3). A low PNI score significantly increased the likelihood of ETI (adjusted odds ratio [aOR]: 0.92, 95% confidence interval [CI]: 0.87–0.99). The occurrence of grade 2 or higher ADRs was another strong predictor (aOR: 5.65, 95% CI: 2.45–15.00).

**Table 3:** Risk factors for early treatment interruption in patients with MAC-PD.

Characteristics	Multivariate	
	aOR	*P* value
Age, years	(0.98–1.04)	0.527
Sex, Male	0.95 (0.46–1.97)	0.895
BMI (kg/m2)	1.12 (1.01–1.24)	0.033
Malignancy	1.20 (0.58–2.48)	0.621
Hb, at treatment initiation	0.85 (0.67–1.09)	0.210
PNI, at treatment initiation	0.92 (0.87–0.99)	0.017
Adverse drug reactions, grade 2 or higher	5.65 (2.45–15.00)	<0.001

aOR=adjusted odd ratio, BMI = body mass index, Hb = hemoglobin, PNI = prognostic nutritional index, MAC-PD = *Mycobacterium avium* complex pulmonary disease.

### Treatment outcome trajectory of ETI and association with all-cause-mortality

**Fig 2:**
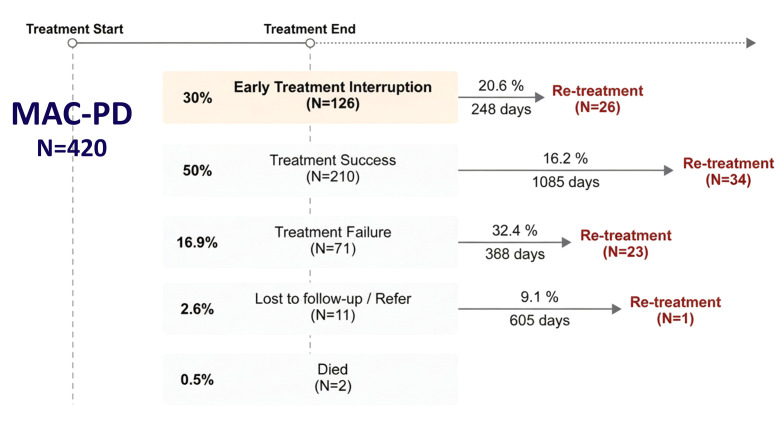
Treatment outcome trajectory in patients with MAC-PD.

During the median follow-up period of 4.9 years (2.5–8.0 years), 20.6% (N = 26) of patients in the ETI group required retreatment for MAC-PD, with a median time to retreatment of 248 days. In contrast, among patients who achieved treatment success (N = 210), retreatment was initiated in 16.2% (N = 34) with a median time to retreatment of 1,085 days (Fig 2). Within the ETI group, among patients who required retreatment, ETI recurred in 38.5% (N = 10), and the treatment success rate was only 19.2% (N = 5) (S2 Table).

**Fig 3:**
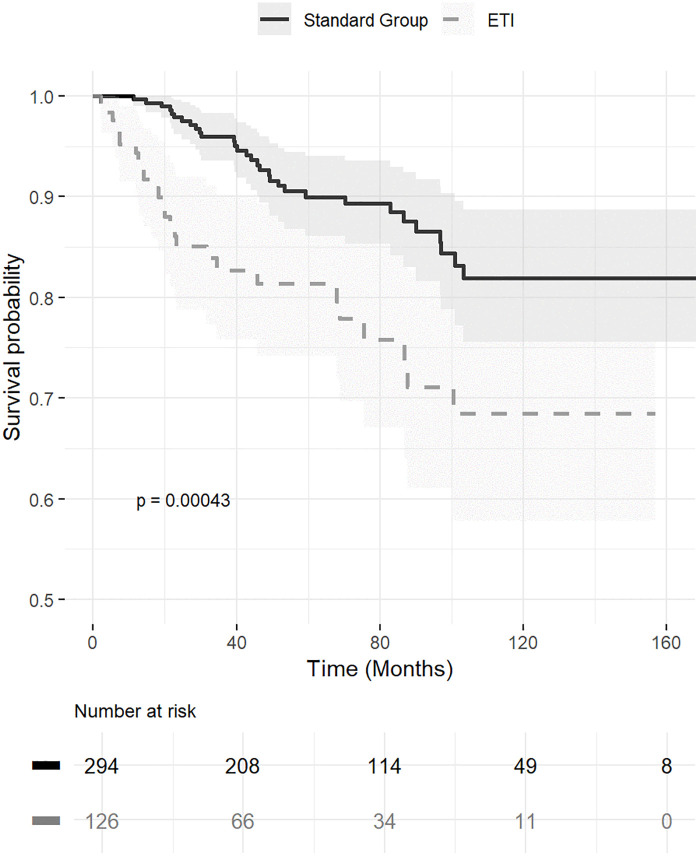
Kaplan–Meier survival curve by completion of treatment.

Kaplan–Meier survival analysis demonstrated significantly worse long-term survival in the ETI group (Fig 3). In multivariate Cox-regression analysis, ETI was identified as an independent risk factor for increased mortality (adjusted hazard ratio, 2.86; 95% CI, 1.51–5.40). Additional factors associated with increased mortality included male sex, positive AFB smear, the comorbidity of malignancy, low PNI levels (Table 4).

**Table 4:** Mortality-associated risk factors in patients with MAC-PD.

Characteristics	Total	Died	Mortality rate	Multivariate	
	(N = 363)	(N = 57)		aHR	*P* value
Age, > 65 years	171	44	25.7	1.87 (0.91–3.87)	0.090
Sex, male	113	44	38.9	2.91 (1.26–6.74)	0.013
BMI, < 18.5 kg/m^2^	83	34	41.0	4.97 (2.67–9.24)	<0.001
Ever smoker	73	30	41.1	1.49 (0.72–3.11)	0.285
TB history	134	35	26.1	1.65 (0.90–3.01)	0.104
Diabetes	42	13	31.0	1.27 (0.61–2.65)	0.516
ILD	6	2	33.3		
COPD	40	18	45.0	1.14 (0.57–2.31)	0.708
Asthma	24	4	16.7		
Malignancy	52	23	44.2	2.41 (1.33–4.37)	0.004
Cardiovascular disease	44	11	25.0		
Chronic kidney disease	16	3	18.8		
Connective tissue disease	18	2	11.1		
Chronic liver disease	18	7	38.9		
Any symptom	279	43	15.4		
AFB smear, Positive	74	20	27.0	2.39 (1.23–4.66)	0.011
Cavity in radiology	124	24	19.4	0.89 (0.47–1.68)	0.718
PNI < 45, at diagnosis	45	23	51.1	1.94 (1.04–3.62)	0.036
Culture conversion	226	19	8.4	0.96 (0.48–1.93)	0.917
ETI	100	26	26.0	2.86 (1.51–5.40)	0.001

aHR = adjusted hazard ratio, BMI = body mass index, TB = tuberculosis, ILD = interstitial lung disease, COPD = chronic obstructive lung disease, AFB = acid fast bacillus, PNI = prognostic nutrition index, ETI = early treatment interruption, MAC-PD = *Mycobacterium avium* complex pulmonary disease.

## Discussion

In this study, approximately 30% of patients with MAC-PD discontinued the treatment early, defined as cessation of therapy within 12 months. ETI was markedly associated with the occurrence of ADRs and poor nutritional status, as indicated by low PNI scores. Furthermore, ETI and poor nutritional status emerged as significant risk factors for increased long-term mortality, highlighting their critical impact on treatment outcomes in patients with MAC-PD.

Optimal treatment adherence beyond 12 months is essential for achieving favorable outcomes in MAC-PD. However, our findings revealed a substantial burden of treatment intolerance, primarily driven by ADRs. The prevalence of ADRs was notably high in the ETI group, with an incidence of 88.1%, and a majority of these ADRs were grade 2 or higher. These findings align with previous studies that highlighted the adverse effects of prolonged multidrug therapy in NTM-PD treatment [18–20]. Given that ADRs were the predominant reason for ETI, early detection, monitoring, and proactive management of ADRs are crucial for improving treatment adherence and outcomes.

Consistent with previous findings [21], our study revealed that 68.8% of patients treated for MAC-PD experienced ADRs. Despite this high prevalence, standardized frameworks for the systematic monitoring and clinical management of ADRs in NTM-PD remain notably limited.

In oncology, frameworks such as the CTCAE [16] and its patient-reported version (PRO-CTCAE) have significantly improved ADR management [22,23], enabling tailored interventions such as dose modifications while preserving therapeutic efficacy [24]. Similarly, the World Health Organization has implemented active tuberculosis (TB) drug safety monitoring and management guidelines for drug-resistant TB [25], providing a structured approach to ADR surveillance and intervention. In contrast, NTM-PD treatment currently lacks a comparable framework, limiting both clinical and research advancements. Furthermore, ADRs can significantly impact patients’ quality of life [26], which is a critical patient-reported outcome in the managing MAC-PD. As demonstrated in our results, it is crucial to implement proactive and systemic monitoring and management of ADRs, which frequently occur during treatment. This should include scheduled functional assessments, such as audiometry for ototoxicity and visual acuity testing for ocular toxicity.

Furthermore, proactive nutritional monitoring and support in high risk patients are essential to ensure optimal treatment adherence. Another key factor influencing treatment adherence in our study was patients’ nutritional status. Previous research has identified malnutrition as a key factor in NTM-PD progression and mortality [27,28], and our findings further emphasize the importance of maintaining adequate nutritional status both at diagnosis and throughout the course of treatment. Among various tools available for assessing nutritional status, the PNI is cost-effective and easily measurable option. The PNI reflects both nutritional and immune status [29], and prior studies have also shown its association with mortality in patients with NTM-PD [30,31]. Low PNI reflects both poor nutritional status and impaired immune competence. Hypoalbuminemia may influence drug pharmacokinetics by reducing protein binding, thereby potentially increasing the risk of drug toxicity [32]. In parallel, lymphopenia may indicate diminished immune resilience and impaired recovery from tissue injury [33,34]. These factors may collectively reduce physiological reserve, rendering patients more vulnerable to the clinical impact of adverse drug reactions and increasing the likelihood of treatment interruption.

Therefore, patients with low PNI may have reduced the tolerance to the cumulative pharmacological burden and multi-organ ADRs associated with long-term MAC-PD therapy. Consequently, even relatively mild ADRs can trigger significant functional deterioration in these vulnerable patients. Given that some patients with NTM-PD are managed conservatively under a watchful waiting strategy [35,36], nutritional assessment and timely interventions remain crucial, even in the absence of active treatment initiation. For patients at-risk, early supportive measure – such as high-protein oral nutritional supplements and structured dietary counseling focus on small, frequent, protein-rich meals – may help preserve physiological reserve and improved treatment tolerance.

Addressing these challenges requires a multidisciplinary approach involving pulmonologists, infectious disease specialists, pharmacists, dietitians, and clinical psychologists [37,38]. This approach should prioritize early identification and management of ADRs, nutritional optimization, and patient education to enhance treatment adherence. Evidence from other chronic diseases suggests that comprehensive multidisciplinary interventions can improve outcomes [39–41], suggesting potential benefits for managing MAC-PD as well.

To the best of our knowledge, this study is one of the first to comprehensively examine the factors associated with ETI and its long-term outcomes in patients with MAC-PD. The findings provide valuable insights into the clinical significance of ADRs and malnutrition in treatment adherence and survival. However, several limitations should be considered. First, as a single-center retrospective study, our findings may not fully represent the heterogeneous spectrum of patients with MAC-PD across different regions and healthcare settings. Second, further investigation is needed to better understand the long-term outcomes of patients who experienced ETI, including the frequency of retreatment, the factors influencing retreatment success or failure, and the overall prognosis following retreatment. Third, lower-grade ADRs (grade 1 and 2) may have been underreported in the retrospective analysis, potentially underestimating their impact on treatment adherence.

## Conclusion

This study highlights the significant burden of ETI in MAC-PD, primarily driven by ADRs and poor nutritional status. Both factors were also associated with increased long-term mortality. These findings underscore the urgent need for enhanced multidisciplinary strategies focusing on ADR management, nutritional support, and treatment adherence to optimize clinical outcomes in patients with MAC-PD.

## Supporting information

S1 Table Treatment related clinical characteristic of patient with MAC-PD based on treatment duration. (DOCX)

S2 Table Re-treatment outcomes in study population. (DOCX)

S3 Table Risk factors for early treatment interruption in patients with MAC-PD (Univariate results). (DOCX)

S4 Table Mortality-associated risk factors in patients with MAC-PD (Univariate results). (DOCX)

S1 Fig 12-Month treatment continuation rate. (TIFF)

## Abbreviation

AFB: Acid-fast bacillus
ADRs: Adverse drug reactions
aOR: Adjusted odds ratio
BMI: Body mass index
CI: Confidence interval
CTCAE: Common Terminology Criteria for Adverse Events
ETI: Early treatment interruption
LFTU: Lost to follow-up
MAC-PD: *Mycobacterium avium* complex pulmonary disease
NTM-PD: Nontuberculous mycobacterium pulmonary disease
PNI: Prognostic Nutritional Index
TB: Tuberculosis
