# Supplementary material for: Early treatment interruption and nutritional status as predictors of mortality in Mycobacterium avium complex pulmonary disease
Source: PLoS One. 2026 May 27;21(5):e0350106. doi: 10.1371/journal.pone.0350106 (PMC13215541; doi:10.1371/journal.pone.0350106)
Supplement: S1 Table — (DOCX) [file pone.0350106.s001.docx]

**eTable 1. Treatment Related Clinical Characteristic of Patient with MAC-PD Based on Treatment Duration**

| Characteristics | Total  (N=420) | Standard group  (N=294) | ETI  (N=126) | *P* value |
| --- | --- | --- | --- | --- |
| Treatment regimen |  |  |  | 0.704 |
| Daily | 303 (72.1) | 210 (71.4) | 93 (73.8) |  |
| Three time weekly | 117 (27.9) | 84 (28.6) | 33 (26.2) |  |
| Triple drug regimen | 398 (94.8) | 282 (95.9) | 116 (92.1) | 0.104 |
| Macrolide/EMB/RIF  (± amikacin) | 361 (86.0) | 260 (88.4) | 101 (80.2) |  |
| Macrolide/EMB/CFZ | 17 (4.0) | 11 (3.7) | 6 (4.8) |  |
| Others^a^ | 20 (4.8) | 11 (3.7) | 9 (7.1) |  |
| Two drug regimen |  |  |  |  |
| Macrolide/RIF or EMB | 22 (5.2) | 12 (4.1) | 10 (7.9) |  |
| IV or IM antibiotics use | 73 (17.4) | 52 (17.7) | 21 (16.7) | 0.911 |
| Lung resection during treatment | 33 (7.9) | 21 (7.1) | 12 (9.5) | 0.527 |
| Treatment duration (days) | 407 (300–536) | 465 (402–580) | 116 (33–240) | <0.001 |
| Culture conversion | 245 (58.3) | 215 (73.1) | 30 (23.8) | <0.001 |
| Time to culture conversion (days) | 50 (35–112) | 55 (35–112) | 45 (28–90) | 0.129 |
| Follow up duration (months) | 58.7  (30.4–97.0) | 66.9  (37.3–97.5) | 45.3  (19.8–86.8) | <0.001 |
| ADRs during treatment | 289 (68.8) | 178 (60.5) | 111 (88.1) | <0.001 |
| ADRs, grade 2 or higher | 214 (51.0) | 112 (38.1) | 102 (81.0) | <0.001 |
| Time to identify ADRs (days) | 54 (20–157) | 78 (29–217) | 36 (14–100) | <0.001 |
| ADRs in multiple organs | 78 (18.6) | 41 (13.9) | 37 (29.4) | <0.001 |

ETI=early treatment interruption, RIF=rifampicin, EMB=ethambutol, IV=intravenous, IM=intramuscular, CFZ= clofazimine, MAC-PD=*Mycobacterium avium* complex pulmonary disease, ADRs=adverse drug reactions.

^a^Others include two-drug regimen with fluoroquinolone or two-drug regimen with nebulized or IV amikacin.
