# Supplementary material for: Early treatment interruption and nutritional status as predictors of mortality in Mycobacterium avium complex pulmonary disease
Source: PLoS One. 2026 May 27;21(5):e0350106. doi: 10.1371/journal.pone.0350106 (PMC13215541; doi:10.1371/journal.pone.0350106)
Supplement: S2 Table — (DOCX) [file pone.0350106.s002.docx]

**eTable 2. Re-treatment Outcomes in Study Population**

| Characteristics | Total | Standard group | ETI group | *P-*value |
| --- | --- | --- | --- | --- |
|  | (N = 420) | (N = 294) | (N = 126) |  |
| Re-treatment | 84 (20.0) | 58 (19.7) | 26 (20.6) | 0.936 |
| Time to re-treatment | 605 (236–1272) | 869 (345–1366) | 248 (130–658) | 0.001 |
| Re-treatment outcome |  |  |  | 0.428 |
| Treatment success | 21 (25.0) | 16 (27.5) | 5 (19.2) |  |
| Treatment failure | 22 (26.2) | 17 (29.3) | 5 (19.2) |  |
| Early treatment interruption | 25 (29.7) | 15 (25.9) | 10 (38.5) |  |
| LTFU or refer | 9 (10.7) | 4 (6.9) | 5 (19.2) |  |
| Ongoing treatment | 7 (8.3) | 6 (10.3) | 1 (3.8) |  |

Data are presented as numbers (percentages).

ETI, early treatment interruption; LTFU, loss to follow-up.
