# Supplementary material for: Early treatment interruption and nutritional status as predictors of mortality in Mycobacterium avium complex pulmonary disease
Source: PLoS One. 2026 May 27;21(5):e0350106. doi: 10.1371/journal.pone.0350106 (PMC13215541; doi:10.1371/journal.pone.0350106)
Supplement: S3 Table — (DOCX) [file pone.0350106.s003.docx]

**eTable3. Risk Factors for Early Treatment Interruption in Patients with MAC-PD (Univariate results)**

| Characteristics | Univariate | |
| --- | --- | --- |
|  | OR | *P* value |
| Age, years | 1.03 (1.01–1.05) | 0.003 |
| Sex, Male | 1.46 (0.95–2.23) | 0.083 |
| BMI (kg/m2) | 1.01 (0.95–1.09) | 0.697 |
| Malignancy | 1.73 (1.02–2.89) | 0.038 |
| Hb, at treatment initiation | 0.79 (0.67–0.92) | 0.004 |
| PNI, at treatment initiation | 0.91 (0.87–0.95) | <0.001 |
| Adverse drug reactions, grade 2 or higher | 6.68 (3.32–14.99) | <0.001 |

OR=odd ratio, BMI=body mass index, Hb=hemoglobin, PNI=prognostic nutritional index, MAC-PD=*Mycobacterium avium* complex pulmonary disease.
