# Supplementary material for: Early treatment interruption and nutritional status as predictors of mortality in Mycobacterium avium complex pulmonary disease
Source: PLoS One. 2026 May 27;21(5):e0350106. doi: 10.1371/journal.pone.0350106 (PMC13215541; doi:10.1371/journal.pone.0350106)
Supplement: S4 Table — (DOCX) [file pone.0350106.s004.docx]

**eTable4. Mortality-Associated Risk Factors in Patients with MAC-PD (Univariate results)**

| Characteristics | Univariate | |
| --- | --- | --- |
|  | HR | *P* value |
| Age, >65 years | 4.18 (2.25–7.79) | <0.001 |
| Sex, male | 6.57 (3.54–12.22) | <0.001 |
| BMI, <18.5 kg/m^2^ | 3.89 (2.29–6.60) | <0.001 |
| Ever smoker | 3.89 (2.31–6.55) | <0.001 |
| TB history | 2.01 (1.18–3.43) | 0.011 |
| Diabetes | 2.26 (1.22–4.20) | 0.010 |
| ILD | 1.61 (0.39–6.67) | 0.511 |
| COPD | 3.14 (1.80–5.49) | <0.001 |
| Asthma | 1.20 (0.43–3.31) | 0.729 |
| Malignancy | 3.31 (1.95–5.62) | <0.001 |
| Cardiovascular disease | 1.65 (0.85–3.16) | 0.144 |
| Chronic kidney disease | 1.08 (0.34–3.47) | 0.893 |
| Connective tissue disease | 0.69 (0.17–2.82) | 0.604 |
| Chronic liver disease | 1.93 (0.88–4.26) | 0.104 |
| Any symptom | 0.97 (0.53–1.77) | 0.922 |
| AFB smear, Positive | 2.86 (1.64–4.98) | <0.001 |
| Cavity in radiology | 1.55 (0.91–2.62) | 0.104 |
| PNI<45, at diagnosis | 4.70 (2.74–8.08) | <0.001 |
| Culture conversion | 0.29 (0.17–0.51) | <0.001 |
| Early treatment interruption | 2.48 (1.47–4.17) | <0.001 |

HR= hazard ratio, BMI=body mass index, TB=tuberculosis, ILD=interstitial lung disease, COPD=chronic obstructive lung disease, AFB=acid fast bacillus, PNI=prognostic nutrition index, MAC-PD=*Mycobacterium avium* complex pulmonary disease.
