# Supplementary figures and images for: Early treatment interruption and nutritional status as predictors of mortality in Mycobacterium avium complex pulmonary disease
Source: PLoS One. 2026 May 27;21(5):e0350106. doi: 10.1371/journal.pone.0350106 (PMC13215541; doi:10.1371/journal.pone.0350106)

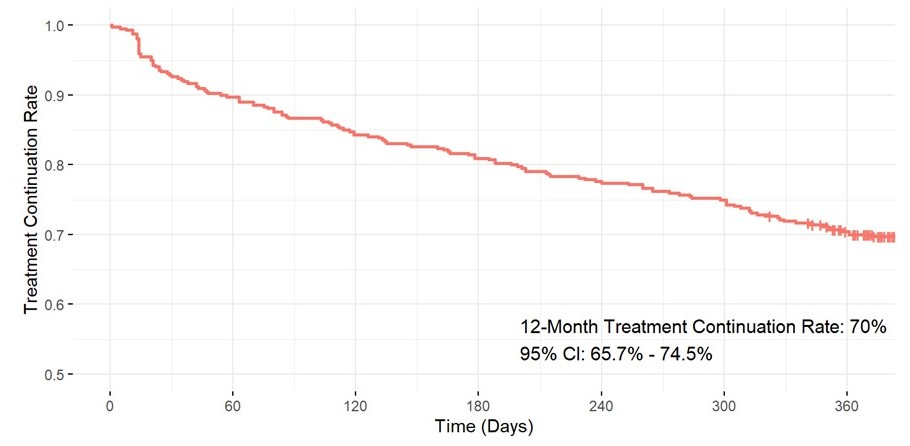

Supplement: S1 Fig — (TIFF) [file pone.0350106.s005.tiff]
